# Investigating the mediating role of food involvement in the relationship between eating restrictions, nutritional knowledge, and dietary patterns in adults

**DOI:** 10.1371/journal.pone.0301533

**Published:** 2024-04-01

**Authors:** Kiyana Saadati, Mohammad Reza Kordbageri, Fakhreddin Chaboksavar, Khadije Jahangasht Ghoozlu, Shabnam Parvizi, Abbas Shamsalinia, Reza Ghadimi, Zeinab Porasgari, Fatemeh Ghaffari

**Affiliations:** 1 Department of Medicine, Mazandaran University of Medical Sciences, Ramsar, Mazandaran, Iran; 2 Department of Statistics and Mathematics, Shahid Beheshti University, Tehran, Iran; 3 Department of Nursing, Babol University of Medical Sciences, Babol, Mazandaran, Iran; 4 Department of Community Medicine, Babol University of Medical Sciences, Babol, Mazandaran, Iran; 5 Department of Nutrition, Islamic Azad University, Tehran, Iran; Flinders University, AUSTRALIA

## Abstract

People’s Dietary Patterns (DPs) are influenced by culture and ethnicity, and their identification requires a holistic assessment of diet. DP reflects dietary behaviors, and its analysis may provide further details about the dietary etiology of chronic diseases. By examining people’s DP and related factors, more practical solutions can be proposed to prevent overweight, obesity, and related diseases. This study aimed to describe DP, Eating Restrictions (ER), Food Involvement (FI), Nutrition Knowledge (NK), and anthropometric indices in Iranian adults and determine the mediating role of FI in the relationship between ER, NK, and DP. A descriptive cross-sectional study was conducted using the structural equation modeling approach. The study was conducted on 2421 adults in Mazandaran Province, northern Iran. The Eating Restrictions Questionnaire (ERQ), the Food Involvement Inventory (FII), the komPAN questionnaire, and a demographic characteristics and anthropometric indices questionnaire were used to collect data. We also measured the seven major food groups, the Diet Quality Scores (DQS), and the six dietary indices, including the pro-Healthy-Diet-Index (pHDI-15), non-Healthy Diet-Index (nHDI-16), high-Glycemic-Diet-Index-7 (hGIDI-7), low-Glycaemic-Diet-Index-4 (lGIDI-4), high-Sugar-Diet-Index-4 (hSDI-4), and high-sugar product (hSFDI-6) and compared their values by gender and four BMI groups. The prevalence of ER and FI was 6.25% and 49.1%, respectively. NK was insufficient for 43.1% of the participants. Most participants (71.2%) had low DQS scores on pHDI-15 and moderate scores (52.9%) on nHDI-16. DQS scores were low in 64.8% of participants in the lGIDI-4 food group, while 71.7%, 92.6%, and 77.2% possessed moderate scores in the hGIDI-7, hSFDI-6, and hSDI-4 food groups, respectively. The mean pHDI-15 and lGIDI-4 indices were higher in women than in men. The amount of unhealthy food indices (nHDI-16, hGIDI-7, hSDI-4, and hSFDI-6) was higher in lean, overweight, and obese people than in people with a normal BMI. The structural model assumed between ER and DP and the mediating role of FI fit well with Iranian adults. Moreover, FI had a mediating role in the relationship between NK and pHDI_15 (Indirect Effects = 0.05, P<0.05) and nHDI_16 (Indirect Effects = -0.07, P<0.01). Most participants are small portions of the healthy food groups and the low glycemic foods, and FI plays a mediating role in the relationship between NK and ER with DP. Therefore, it is necessary to pay attention to the role of FI as a mediating variable in interventions based on reducing ER, increasing NK, and shifting DP from unhealthy to healthy.

## Introduction

Dietary patterns (DPs) in low- and middle-income countries have changed significantly in recent years [1]. Most countries are undergoing a nutrition transition and are struggling with a growing burden of malnutrition [2]. DP refers to the amount, proportion, variety, or combination of different foods, drinks, and nutrients in the diet and how often they are typically consumed [3]. DP varies by country, culture, or ethnicity, and its identification necessitates a holistic dietary assessment [4]. Studies have shown that an unhealthy diet can lead to diet-related chronic diseases such as obesity, diabetes, cardiovascular diseases, and some cancers [5].

Identifying and describing the DP and its related factors can lead to effective measures to prevent the consequences of unhealthy DPs [6]. Eating restriction (ER) is one of the factors that influence DP [7]. Studies have shown that a significant portion of people have some ERs [8, 9]. ER is often rooted in dissatisfaction with body weight and shape, allergies [10], religious and ideological beliefs [11], and has positive and negative effects on people’s nutritional status [8, 12, 13], health, and quality of life [14]. For instance, it may lead to unhealthy eating habits [15] or cause psychological consequences, such as preoccupation with food and eating, increased emotional responsiveness, dysphoria, and distractibility [16]. Therefore, ER serves as a predictor of people’s nutritional status and health [15].

Food involvement (FI) is another factor associated with DP [17]. Rozin et al. (1999) explored the concept of FI in their study [18]. Bell and Marshall defined FI as "the importance that food has in a person’s life" [19]. It is believed that a person’s involvement with food and the time that he/she invests in preparing food play an important role in adopting healthy eating habits [20]. According to Laaksonen, involvement is a mediating variable in the relationship between stimulus (food) and response [21]. The three categories of involvement include enduring involvement (a person’s long-term attachment to a specific product or advertisement), situational involvement (a person’s involvement and concern in purchasing a particular product), and response involvement (the behavioral orientation that includes information gathering and the decision-making process) [22]. FI is a multifactorial and multidimensional phenomenon that involves the consumer in a 360-degree way and deals with social, cognitive, emotional, and psychological dimensions related to identity [22–24].

Nutritional knowledge (NK) is also one of the factors influencing healthy food choices, appropriate DP, and the maintenance of a healthy diet [25]. Several studies have highlighted the effects of NK on diet-related behaviors and DP [26, 27]. NK refers to being aware of practices and concepts related to nutrition and health, such as adequate food intake, the impact of foods and food choices on diseases, the list of foods with major sources of nutrients, and dietary guidelines and recommendations related to a healthy diet [26].

Since DP is a leading cause of chronic diseases, a thorough understanding of DP is required to develop strategies to reduce diet-related disorders at the national and global levels [28]. In recent years, much attention has been given to the analysis of DP and related factors in different groups, including adults. The American Dietetic Association suggests that healthy eating messages to the public should focus on DP rather than foods or meals [29]. Assessing DP provides a more comprehensive picture of people’s dietary behaviors, identifies diet-related causes of chronic diseases, and offers practical solutions to prevent overweight, obesity, and related diseases [30]. It also helps identify the combined effects of commonly consumed foods and people’s dietary interests and preferences based on their cultural, economic, and social structure and lifestyle [31–33]. However, there is limited evidence regarding the DP and the factors influencing it, especially in Iran. Existing studies have also examined limited variables and have not clarified which variables might play a moderating role in the relationship between independent factors and DP. A study reported that FI could be an important mediator to consider when studying diet and eating habits [19]. Therefore, the present study tried to answer the question of whether FI can mediate the effects of ER and NK on DP.

### Objectives

This study aimed to describe DP, ER, FI, NK, and anthropometric indices in Iranian adults and to determine the mediating role of FI in the relationship between ER, NK, and DP.

### Hypotheses

- There is a significant relationship between ER and DP in adults.
- There is a significant relationship between NK and DP in adults.
- FI mediates the relationship between ER and DP in adults.
- FI mediates the relationship between NK and DP in adults.
- The structural equation explaining the mediating role of FI in the relationship between ER, NK, and DP has a good fit.

**Fig 1:**
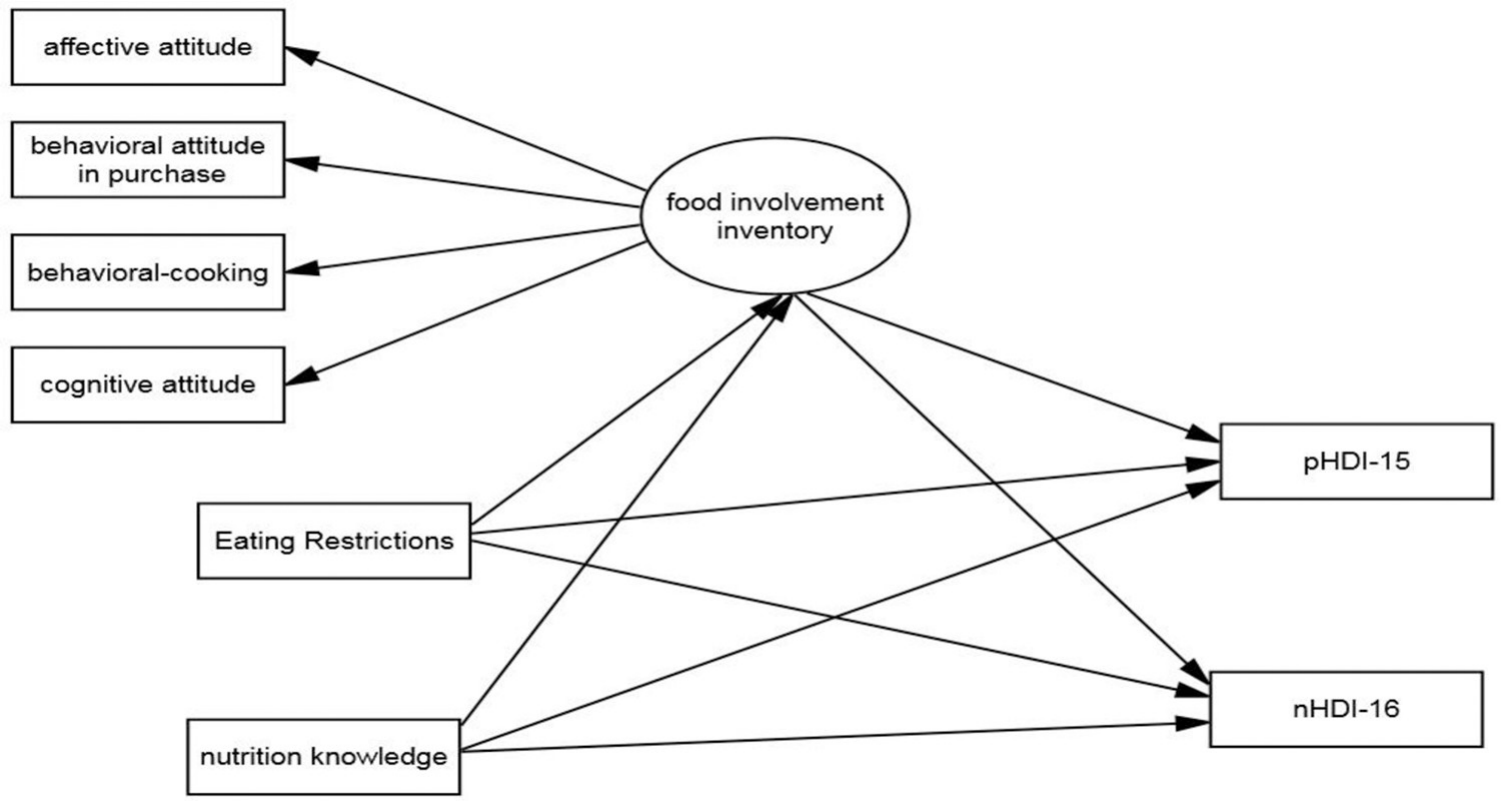
The conceptual model.

Based on the work done by Jezewska-Zychowicz [34], we developed the following conceptual model (Fig 1).

## Materials and methods

### Design and setting

A descriptive cross-sectional study was conducted using a structural equation modeling technique. The study was conducted during 2022 and 2023 on a convenient sample of people who referred to the comprehensive health service centers in western Mazandaran province, northern Iran. This study is part of a project under the code 724133802.

### Population

We conducted a pilot study on 100 adults to determine the sample size. Sampling was started after receiving the needed permissions. Given a low NK probability of 70% (p = 0.7), a type 1 error of 0.05, and a measurement error of 0.02 (d = 0.02), we estimated that 2017 subjects needed to be recruited for the study. However, considering a possible dropout of 20%, the sample size was increased to 2421.

Inclusion criteria included age between 18 and 60 years, ability to read and write in Persian, lack of known chronic disorders (self-reported), lack of a specific diet, lack of allergies, and lack of a specific meal plan. A complete non-response to the questionnaires and a decision to withdraw from the study were considered the exclusion criteria.

### Measures

#### Eating Restrictions Questionnaire (ERQ)

This questionnaire was originally developed by Jezewska-Zychowicz et al. and measures 10 categories of eating restrictions [34]. The 13-item, Persian version of this questionnaire was validated in the Iranian adult population (internal consistency = 0.865) [35].

#### Food Involvement Inventory (FII)

This 25-item scale was developed by Yun-Mi Lee et al. and includes four subscales, namely the affective, cognitive, behavioral-purchase, and behavioral-cooking [36]. This instrument has already been translated into Persian, and the number of its items was reduced to 24 after removing the item "When I buy food, I check the information on the package." This inventory has acceptable validity and reliability (Cronbach’s alpha coefficient = .786) for Iranian adults [35].

#### Dietary Patterns (DPs) assessment scale

In this study, we used the self-report version of the komPAN questionnaire to measure DP [37]. The komPAN questionnaire has four parts: Dietary Habits (DH), Frequency of Food Consumption (FFC), Nutrition Knowledge (NK), and Lifestyle. In the present study, the FFC subscale was used to measure DP. The FFC has 33 items to assess the seven main dietary patterns, including grain products (4 items), fruits/vegetables/legumes/potatoes (5 items), dairy products (4 items), meat/fish/eggs (6 items), fats (3 items), beverages (7 items), sweets, and other products (4 items).

Respondents indicate their FFC on a 6-point Likert scale from never, 1–3 times a month, once a week, several times a week, once a day, or several times a day. For frequency of consumption (times/day), numerical values are assigned to FCC (once a day = 1, few times a day = 2, few times a week = 0.5, once a week = 0.14, 1–3, times a month = 0.06, and never = 0). The psychometric indices of all parts of the komPAN questionnaire have been studied in the Iranian adult population, and the relevant details are provided in our previously published article [38].

DP can be extracted using a priori (hypothesis-based) or a posteriori (data-based) approach [39, 40]. The hypothesis-based DP (Diet Quality Scores) is derived based on dietary recommendations and reflects adherence to healthy or unhealthy eating habits [41–43]. Data-based DP is identified using statistical methods such as cluster analysis (CA), principal component analysis (PCA), and explanatory factor analysis (EFA) [44]. In this study, we used the first approach to examine the food indices hSFDI-6, hSDI-4, lGIDI-4, hGIDI-7, and the second approach to examine the food indices 16-pHDI-15 and nHDI.

#### Diet Quality Scores

We measured six dietary indices, including pro-Healthy-Diet-Index (pHDI-15), non-Healthy Diet-Index nHDI-16, hGIDI-7, lGIDI-4, hSDI-4, and hSFDI-6 [38]. The values of these indices were compared based on gender and four BMI (kg/m^2^) categories (i.e., thin: BMI<18.5, normal: BMI = 18.5–24.9, overweight: BMI = 25–29.9, and obese: BMI ≥30) [45]. We also calculated the diet quality scores (DQS) using the following formula, where A is the total daily intake of all foods in a food group and B represents the total maximum possible daily intake of the same foods [46]. Food groups consumption levels (indices) are reported as a percentage and are classified as low (0–33·32%), moderate (33·33–66·65%), and high (66·66–100%), indicating the intensity of consumption of selected food groups [46].


$$Diet\;Quality\;Index=\frac{100\%*\sum A}{\sum B}$$

#### Other variables

*Demographic profile*. This questionnaire includes items on participants’ age, sex, education level, marital status, place of residence, occupation, household partner, financial status, and the number of people in the household.

*Anthropometric indices*. We also measured the height, weight, body mass index, waist circumference (WC) (≤ 102 cm for men and ≤ 80 cm for women is considered normal [47], hip circumference (HC), waist-to-hip ratio (WHR), and waist-to-height ratio (WHtR ((an indicator for obesity). Participants’ weight was measured with minimal clothing, no shoes, and using a digital scale (ASMOD^®^, Iran, precision weighting of ±100 gr) after calibration. Height, HC, and WC were also measured using a rigid plastic tape measure with a precision of 0.1 cm. A qualified research assistant performed all anthropometric measurements.

WC was measured at the level of the navel, and HC at its most prominent point. WHR was calculated by dividing WC by HC, and WHtR was calculated by dividing WC by height. The proposed optimal cutoff values for WC, WHtR, and WHR were 84 cm, 0.48 and 0.78 for women and 98 cm, 0.56 and 0.87 for men, respectively [48]. BMI was also categorized as thin (≤18.5), normal (18.5–24.9), overweight (25–29.9), and obese (≥ 30) [45].

*Nutrition Knowledge (NK)/ Nutritional Beliefs*. To assess NK, we used the NK section of the self-report version of the komPAN questionnaire [37].This scale consists of 25 items, each rated on a 3-point Likert scale (true = 1, false = 0, and not sure = 0). The scores of the items are added up, and the total score ranges between 0 and 25. Scores 0–8, 9–16, and 17–25 are considered insufficient, sufficient, and good NK, respectively [49]. This scale demonstrated acceptable validity and reliability (ICC = 0.917) for the Iranian adult population [38].

#### Statistical analyses

AMOS_24_ software was used to perform structural equation modeling and evaluate the proposed model of the relationship between ER and NK with DP and the mediating role of FI as well as the moderating role of sex.

To evaluate model fit, we used the comparative fit index (CFI)> 0.9, the incremental fit index (IFI)> 0.9, the goodness of fit index (GFI)> 0.9, the parsimonious comparative fit index (PCFI)> 0.5, the parsimonious normed fit index (PNFI)> 0.5, and the root mean square error of approximation (RMSEA) < 0.08.

As suggested by Kline (2023), the skewness and kurtosis values were assessed to check the assumption of univariate normality before conducting the structural equation method and testing the research hypotheses [50]. The skewness and kurtosis values of the variables were in the range of ±2, confirming the univariate normality of the variables. Mardia’s standardized kurtosis coefficient and critical ratio (CR) were also used to check the multivariate normality. According to Blunch (2012), CR values < 5 confirm multivariate normality [51]. In this study, the Mardia coefficient was 3.011 and the CR was 2.260; therefore, the assumption of multivariate normality is maintained. The Mahalanobis distance index (d-squared method) was examined at a significance level >0.5 to ensure the absence of multivariate outlier data. No outliers were detected in this study.

### Ethics statement

To conduct the present study, permission was received from the ethics committee of the Babol University of Medical Sciences (IR.MUBABOL.HRI.REC.1400.046). A written informed consent form was signed by all participants, before participation. The objectives of the study were explained to all participants, and all had the right to withdraw from the study. They were assured that the principle of confidentiality would be respected.

## Results

The majority of participants (53.9%) were women, and 60.2% were overweight or obese. The frequency of obesity and overweight was higher in men (63.3%) than in women (57.5%) (P<0.001). The mean age (P < .001), height (P<0.001), weight (P<0.001), and WC (P = 0.002) were higher in men than in women. The mean HC was higher in women than in men (P = 0.002). The WHR was higher in men than in women, while the WHtR was higher in women than in men (P<0.001) (Table 1).

**Table 1:** Demographic characteristics of the participants by gender.

Variables		Full sample (N = 2401)		Male (n = 1108)		Female (N = 1293)		P
		n	%	n	%	n	%	
Marital status	Married	1380	57.5	614	55.4	766	59.2	.001^a^
	Single	717	29.9	322	29.1	395	30.5	
	Widowed	162	6.7	90	8.1	72	5.6	
	Divorced	142	5.9	82	7.4	60	4.6	
Occupation	Official	667	27.8	350	31.6	317	24.5	< .001^a^
	Retired	196	8.2	132	11.9	64	4.9	
	Homemaker	633	26.4	43	3.9	590	45.6	
	Self-empolyed	454	18.9	378	34.1	76	5.9	
	Unemployed	215	9	127	11.5	88	6.8	
	Other	236	9.8	78	7	158	12.2	
Education level	Primary education	505	21	247	22.3	258	20	.005^a^
	High school diploma	568	23.7	269	24.3	299	23.1	
	Associate degree	363	15.1	190	17.1	173	13.4	
	Bachlor’s degree	679	28.3	275	24.8	404	31.2	
	Masters’ degree	231	9.6	103	9.3	128	9.9	
	Ph.D	55	2.3	24	2.2	31	2.4	
Financial status	Less than average	628	26.2	344	31	284	22	< .001^a^
	At average	1602	66.7	686	61.9	916	70.8	
	Above average	171	7.1	78	7	93	7.2	
Place of residence	Rural area	476	19.8	246	22.2	230	17.8	.007^a^
	Urban area	1925	80.2	862	77.8	1063	82.2	
Household partner	Alone	255	10.6	154	13.9	101	7.8	< .001^a^
	With spouse	442	18.4	191	17.2	251	19.4	
	With spouse and children	878	36.6	424	38.3	454	35.1	
	With children	152	6.3	56	5.1	96	7.4	
	With family members	674	28.1	283	25.5	391	30.2	
Household size	1–3	1228	51.1	554	50	674	52.1	.422^a^
	4–7	1143	47.6	542	48.9	601	46.5	
	>7	30	1.2	12	1.1	18	1.4	
BMI (kg/m2)	<18.5	49	2	4	.4	45	3.5	< .001^a^
	18.5–24.9	908	37.8	403	36.4	505	39.1	
	25–29.9	880	36.7	434	39.2	446	34.5	
	≥30	564	23.5	267	24.1	297	23	
Age(years); Mean (SD)		37.58(11.30)		38.94(10.87)		36.41(11.53)		< .001^b^
Weight(kg); Mean (SD)		75.22(13.82)		81.30(12.57)		70.01(12.66)		< .001^b^
Height (cm); Mean (SD)		167.90(9.52)		174.15(7.51)		162.55(7.60)		< .001^b^
Waist circumference (cm); Mean (SD)		90.07(14.92)		90.90(14.40)		89.36(15.32)		.012^b^
Hip circumference (cm); Mean (SD)		97.69(14.19)		96.70(14.01)		98.54(14.30)		.002^b^
Waist-to-Hip Ratio; Mean (SD)		.92(.12)		.94(.10)		.91(.14)		< .001^b^
Waist-to-Height Ratio; Mean (SD)		.53(.09)		.52(.08)		.55(.10)		< .001^b^

^a^: *χ*^2^ test

^b^: Independent t Test.

Totally, 6.25% of participants reported ER. In addition, 11.2% and 10.7% of participants reported restrictions on “the quantity of food” and on “high-fat foods,” respectively, but the least restrictions were on the consumption of cereals (5.1%) and dairy products (5.4%). The mean ER score was higher in men than in women (P = 0.030).

Most participants (49.1%) reported FI, such that 63% agreed with the item "I try to buy satisfactory food." The highest mean FI scores were for the “behavioral cooking" and “affective” subscales, with mean scores of 20.34±4.99 and 15.60±3.46, respectively. Women scored higher than men on the FII and all its subscales (P < .001).

Most participants (71.2%) had low DQS scores on healthy foods (pHDI-15) and moderate scores (52.9%) on unhealthy foods (nHDI-16). DQS scores were low in 64.8% of participants in the low GI food group (lGIDI-4), whereas 71.7%, 92.6%, and 77.2% possessed moderate scores in the high Gl food group (hGIDI-7), high saturated fat food group (hSFDI-6), and high-Sugar-Diet-Index-4 food group (hSDI-4), respectively.

On the FCC assessment, the item "How often do you drink water?" received the highest score with a mean of 1.68, while the item "How often do you consume whole cereals such as oats, barley, and wheat (whole or oatmeal), corn, popcorn, and brown rice?" received the lowest score on this scale. Similarly, in the unhealthy foods domain, the items "How often do you use white bread (lavash) or bakery products such as baguettes and toast?" and "How often do you drink alcoholic beverages?" obtained the highest and lowest mean scores (i.e., 0.78 and 0.05, respectively) (Table 2).

**Table 2:** Healthy dietary pattern question/items mean scores and 1st and 3rd quartiles.

No	Items	Mean	Q_25_-Q_75_
	pHDI-15		
2	How often do you consume whole-wheat breads such as homemade bread, barbari, sangak, taftoon, and toast?	.53	.14-.50
4	How often do you consume whole cereals such as oats, barley, and wheat (whole or oatmeal), corn, popcorn, and brown rice?	.25	.06-.50
9	How often do you consume vegetable oils (such as sesame oil, olive oil, sunflower oil, and canola oil), animal butter, and margarine butter for cooking?	.32	.06-.50
10	How often do you drink regular milk (plain milk without additives) or flavored milks such as cocoa milk, coffee milk, date milk, banana milk, honey milk, and strawberry milk?	.33	.06-.50
11	How often do you consume dairy products such as yogurt, buttermilk, curd, and ice cream?	.54	.14-.50
12	How often do you use high-fat cheese?	.38	.06-.50
16	How often do you eat white meat, such as chicken, ostrich, turkey, quail, and partridge, and seafood, such as fish and shrimp?	.37	.06-.50
17	How often do you consume nuts, sunflower seeds, pistachios, hazelnuts, and walnuts?	.39	.06-.50
18	How often do you eat bird eggs such as chicken, quail, partridge, duck, and goose eggs?	.38	.10-.50
19	How often do you eat legumes such as split pea, bean, chickpea,mung bean, lentil, red lentil, broad bean, and soybean?	.33	.06-.50
20	How often do you eat potatoes but not crisps?	.36	.14-.50
21	How often do you eat fruit (raw and dried fruit)?	.60	.14–1
22	How often do you eat raw vegetables (lettuce, carrots, cabbage, pumpkin, onions, mushrooms, cauliflower, broccoli, celery, spinach, vegetables, tomatoes, and cucumbers) and cooked vegetables?	.54	.14-.50
28	How often do you drink vegetable juices or fruit and vegetable juices?	.29	0-.5
32	How often do you drink water?	1.68	0–2
	**nHDI-16**		
1	How often do you use white bread (lavash) or bakery products such as baguettes and toast?	.78	.06–2
3	How often do you use white rice or white pasta?	.52	.14-.5
5	How often do you eat fast food such as French fries, burgers, and pizza?	.26	.06-.5
6	How often do you consume fried foods such as fried meat or fried sweets such as dumplings?	.30	.06-.5
7	How often do you use butter (animal or vegetable) or oils, such as homemade oils, to flavor your bread?	.29	.06-.5
13	How often do you use processed cheese?	.43	.14-.5
14	How often do you consume smoked sausages, cold meats, and hot dogs?	.25	0-.5
15	How often do you consume red meat and offal of livestock and poultry such as heart, liver, gizzard, Kalle pache (Khash), kidney, tripe, and abomasum?	.31	.06-.5
23	How often do you consume sweets such as pastries, biscuits, Sohan, cakes, chocolates, jams, and fruit compotes?	.45	.06-.5
24	How often do you use ready-made or instant soups such as noodle, mushroom, chicken, or vegetable soups?	.27	.06-.5
26	How often do you eat tinned (jar) vegetables such as pickles and peas?	.24	0-.5
27	How often do you drink still beverages?	.34	.06-.5
29	How often do you drink sweetened hot beverages such as black tea, coffee, and herbal or fruit teas?	.71	.14–1
30	How often do you drink sweetened carbonated and noncarbonated drinks like Coca-Cola, Pepsi, Lemonade, or Fanta?	.35	.06-.5
31	How often do you drink energy drinks like Red Bull, Life, Monster, or Rockstar?	.07	0-.12
33	How often do you drink alcoholic beverages?	.05	0-.10

The mean pHDI-15 (P = 0.001) and lGIDI-4 (P < .001) indices were higher in women than in men. The mean NK was higher in men than in women (P = 0.033). Additionally, 43.1%, 43.8%, and 13.2% of participants demonstrated insufficient, sufficient, and good NK, respectively (Table 3).

**Table 3:** Comparison of food indices, nutrition knowledge, eating restrictions, and food involvement by gender.

Variables	Full sample (N = 2401)		Male (N = 1108)		Female (N = 1293)		P
	Mean	SD	Mean	SD	Mean	SD	
**ER (points)**	3.34	3.51	3.51	3.52	3.20	3.50	.030^a^
**FI (points)**	61.56	12.69	57.55	12.03	65.01	12.23	< .001^a^
**BC (points)**	20.34	4.99	18.48	4.75	21.94	4.62	< .001^a^
**A (points)**	15.60	3.46	14.78	3.40	16.31	3.36	< .001^a^
**BP (points)**	13.05	3.03	12.56	3.03	13.46	2.98	< .001^a^
**C (points)**	12.55	2.92	11.71	2.84	13.28	2.79	< .001^a^
**pHDI-15 (%)**	24.61	10.21	23.83	9.70	25.28	10.59	.001^a^
**nHDI-16 (%)**	17.81	8.96	18.07	8.91	17.59	9.01	.196^a^
**hGIDI-7 (%)**	26.98	13.63	27.23	13.84	26.76	13.45	.402^a^
**hSDI-4 (%)**	23.30	16.21	23.88	15.90	22.80	16.46	.104^a^
**hSFDI-6 (%)**	13.66	11.59	13.53	11.32	13.77	11.83	.609^a^
**lGIDI-4 (%)**	20.90	13.29	19.38	11.62	22.21	14.44	< .001^a^
**NK (points)**	10.25	5.18	10.50	5.19	10.04	5.17	.033^a^

ER: Eating Restrictions; FI: Food Involvement; BC: Behavioral Cooking; A: Affective; BP: Behavioral Purchase; C: Cognitive; pHDI-15: pro-Healthy-Diet-Index; nHDI-16: non-Healthy Diet-Index; hGIDI-7: high-Glycaemic-Diet-Index; hSDI-4: high-Sugar-Diet-Index; hSFDI-6: high-Saturated-Fats-Diet-Index; lGIDI-4: low-Glycaemic-Diet-Index; NK: Nutrition Knowledge.

**Fig 2:**
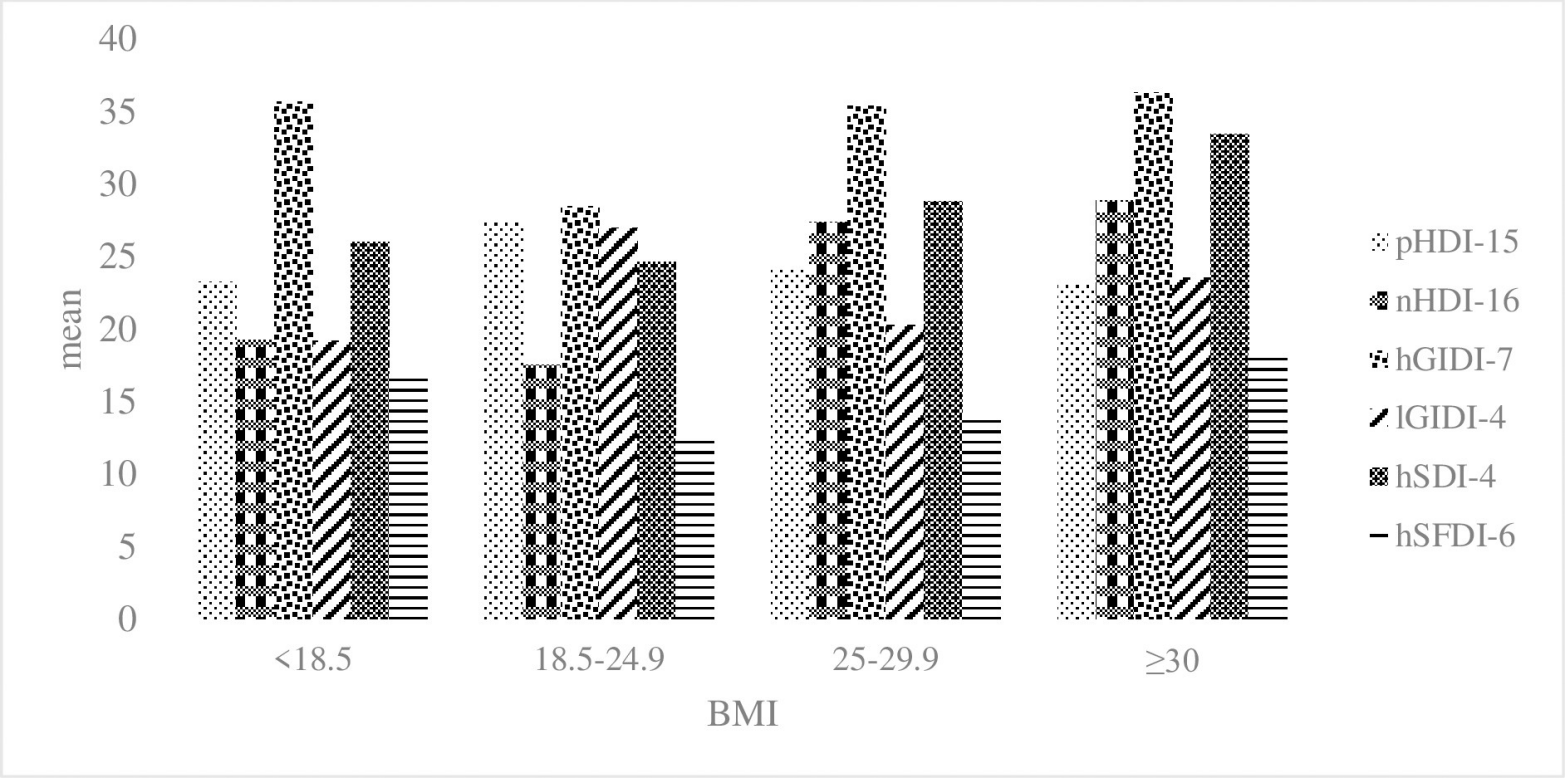
Comparison of nutritional indices by body mass index.

The values of the pHDI-15 and LGIDI-4 indices were higher in subjects with a normal BMI than in others (P<0.001). However, the levels of nHDI-16, hGIDI-7, hSDI-4, and hSFDI-6 were higher in lean, overweight, and obese individuals than in those with a normal BMI (P<0.001) (Fig 2).

According to Pearson`s correlation coefficient, ER was inversely correlated with pHDI-15, NK, and FI and directly correlated with nHDI-16. Furthermore, FI was directly correlated with pHDI-15, and NK, but indirectly with nHDI-16 (Table 4).

**Table 4:** Correlation coefficients and descriptive indices of variables.

	1	2	3	4	5
**1.pHDI-15**	1				
**2.nHDI-16**	-.459**	1			
**3.NK**	.364**	-.300**	1		
**4.FI**	.379**	-.273**	.240**	1	
**5.ER**	-.495**	.293**	-.307**	-.355**	1
**Range**	1.67–90	.81–62.94	0–23	24–96	0–13
**Cronbach’s alpha**	.812	.820	.799	.801	.847
**Skewness**	.967	.708	.432	-.108	.667
**Kurtosis**	1.867	.706	-.349	.505	-.633

Note.

**P < 0.001, N = 2401. ER: Eating Restrictions; FI: Food Involvement; pHDI-15: pro-Healthy-Diet-Index; nHDI-16: non-Healthy Diet-Index; NK: Nutrition Knowledge; M: Mean; SD: Standard Deviation.

The fit indices of the modified model (e.g., χ^2^ = 60.19; RMSEA = .03; CFI = .98; IFI = .98; GFI = .98; PNFI = .59; PCFI = .59) show the acceptable fit of the model and confirm the first hypothesis of the study. That is, the assumed structural model between ER and DP and the mediating role of FI fit well with Iranian adults. The fit indices of the studied model before and after modification are presented in Table 5.

**Table 5:** Fit indices of the primary and modified models.

	χ2	Df	P-Value	CMIN/DF	RMSEA	PNFI	CFI	PCFI	IFI	GFI
**Primary model**	88.17	17	< .001	5.18	.04	.54	.89	.54	.89	.93
**Modified model**	60.19	14	< .001	4.29	.03	.59	.98	.59	.98	.98

*Abbreviations: CMIN/DF: Chi-square/degree-of-freedom ratio; RMSEA: Root Mean Square Error of Approximation; PCFI: Parsimonious Comparative Fit Index; GFI: Goodness of Fit Index; PNFI: Parsimonious Normed Fit Index; IFI: Incremental Fit Index; CFI: Comparative Fit Index.

**Fig 3:**
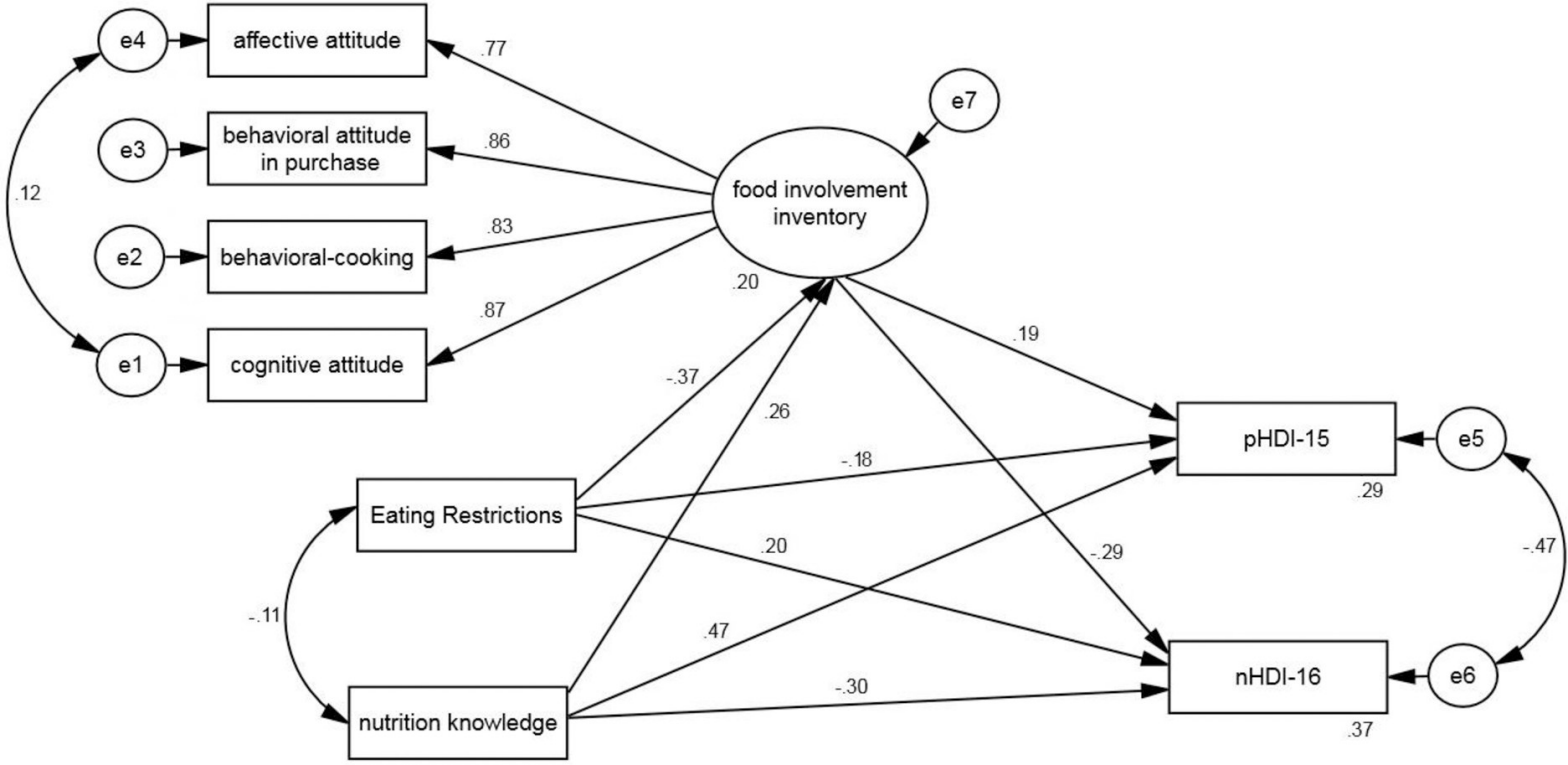
Standardized coefficients of the modified model.

Standardized regression coefficients showed that FI had a direct effect on pHDI_15 (β = 0.19, P<0.001) and an indirect effect on nHDI_16 (β = -0.29, P<0.001). Furthermore, ER had an indirect effect on FI (β = -0.37, P<0.001) and pHDI-15 (β = -0.18, P<0.001) and a direct effect on nHDI-16 (β = 0.20, P<0.001). Also, NK had a direct effect on FI (β = 0.26, P<0.001) and pHDI-15 (β = 0.47, P<0.001) and an indirect effect on nHDI-16 (β = -0.30, P<0.001). These results confirm the second and third hypotheses of the study (Table 5 and Fig 3).

The bootstrap test results suggest that FI plays a mediating role in the relationship between ER and pHDI_15 (Indirect effects = -0.07, P<0.01) and nHDI_16 (Indirect effects = 0.11, P<0.001). Moreover, FI plays a mediating role in the relationship between NK and pHDI_15 (Indirect effects = 0.05, P<0.05) and nHDI_16 (Indirect effects = -0.07, P<0.01). These findings confirm the fourth and fifth hypotheses of the study. In other words, FI plays a mediating role in the relationship between ER and NK with DP in adults (Table 6).

**Table 6:** Standardized coefficients for direct and indirect routes.

Path Estimate	Path Coefficient	S.E.	C.R.	P
**Direct effect**				
ER—> pHDI_15	-.18	.07	-7.23	< .001
ER—>nHDI_16	.20	.10	9.33	< .001
ER—> NK	-.10	.03	-5.04	< .001
ER—> FI	-.37	.15	-23.56	< .001
FI—> pHDI_15	.19	.08	8.74	< .001
FI—>nHDI_16	-.29	.11	-15.32	< .001
FI—> NK	.26	.09	12.04	< .001
**Indirect Effect**	**Path Indirect**	**S.E.**	**Lower**	**Upper**
ER—>FI—>pHDI_15	-.07	.005	-.08	-.06
ER—>FI—>nHDI_16	.11	.01	.09	.13
ER—>FI—>NK	-.10	.01	-.11	-.09

Note. N = 2401. ER: Eating Restrictions; FI: Food Involvement; pHDI-15: pro-Healthy-Diet-Index; nHDI-16: non-Healthy Diet-Index; NK: Nutrition Knowledge.

The model fit indices confirmed the proposed model for both women and men. The results showed that the indirect effect of ER on pHDI_15 through FI was greater in women (β = -0.06, P<0.001) than in men (β = -0.02, P<0.001). In addition, no significant difference was found between the indirect effect of ER on nHDI_16 through FI in men and women (β = 0.03, P<0.001).

The indirect effect of NK on pHDI_15 through FI was also greater in women (β = 0.09, P<0.001) than in men (β = 0.03, P<0.001). Furthermore, the indirect effect of NK on nHDI_16 through FI was greater in women (β = -0.05, P<0.001) than in men (β = -0.04, P<0.001). Comparison of the path coefficients of the two models with and without ER confirmed the moderating role of gender and the mediating role of FI in the relationship between ER and NK with DP (Δχ2(10) = 97.543, P<0.001) (Table 7).

**Table 7:** The moderating effect of gender and the mediating role of food involvement in the relationship between eating restrictions and dietary patterns.

Moderate		Path	Estimate	CMIN/DF	RMSEA	CFI	GFI
Gender	Female	ER—> pHDI_15	-.12*	3.92	.05	.98	.98
		ER—>nHDI_16	.11*				
		ER—> NK	-.19*				
		ER—> FI	-.24*				
		FI—> pHDI_15	.24*				
		FI—>nHDI_16	-.12*				
		FI—> NK	.17*				
		ER—>FI—>pHDI_15	-.06*				
		ER—>FI—>nHDI_16	.03*				
		ER—>FI—>NK	-.04*				
	Male	ER—> pHDI_15	-.09*	4.75	.06	.95	.96
		ER—>nHDI_16	.08*				
		ER—> NK	-.12*				
		ER—> FI	-.20*				
		FI—> pHDI_15	.10*				
		FI—>nHDI_16	-.14*				
		FI—> NK	.12*				
		ER—>FI—>pHDI_15	-.02*				
		ER—>FI—>nHDI_16	.03*				
		ER—>FI—>NK	-.02*				

Note. N = 2401. ER: Eating Restrictions; FI: Food Involvement; pHDI-15: pro-Healthy-Diet-Index; nHDI-16: non-Healthy Diet-Index; NK: Nutrition Knowledge.

## Discussion

This study described the DP, ER, FI, NK, and anthropometric indices in Iranian adults, and investigated the mediating role of FI in the relationship between ER and NK with DP. We examined seven main DPs, including grain products, fruits/vegetables/legumes/potatoes, dairy products, meat/fish/eggs, fats, beverages, sweets, and other products, in Iranian adults. We also measured six food indices, including pro-Healthy-Diet-Index (pHDI-15), non-Healthy Diet-Index (nHDI-16), hGIDI-7, lGIDI-4, hSDI-4, and hSFDI-6. We also compared the values of these indices by four BMI groups, calculated the DQS, and measured the FI, ER, and NK values.

In the current study, 60.2% of participants were overweight or obese, with obesity and overweight being more common in men than in women. A study found that the overall prevalence of normal BMI, obesity, and overweight in Iranian adults was 36.7%, 22.7%, and 59.3%, respectively, which is consistent with our results. However, the prevalence of obesity was higher in women (29.8%) than in men (15.3%) [52], which is inconsistent with our results. Another Iranian study also reported that 42.7% of adults were overweight, 45% were obese, and 71.8% of men and 97.9% of women had high WHR [53]. In the present study, the mean values of WC, WHtR, and WHR in women and only WHR in men were above the normal range, indicating abdominal obesity. Since abdominal obesity is associated with an increased risk of cardiovascular diseases (CVD) and increased WC, WHR, and WHtR levels are good predictors for CVD [54], preventive measures must be taken to reduce risk factors for CVD in adults, especially in women. Totally, 6.25% of our participants reported an ER, with the highest restriction on “the quantity of food" and "on high-fat foods." In a study by Jezewska-Zychowicz et al., almost two-thirds of subjects reported restrictions on the intake of certain foods, either continuously (11.7%) or occasionally (54.7%). Most of the restrictions were for sugar and/or sweets (47.6%), fat (24.9%), and high-fat foods (21.4%) [34]. In another study, nearly one-third of subjects restricted their intake of certain foods, especially the amount of food they ate. Most of the restrictions were related to sugar and/or sweets (23.7%), high-fat foods (22.4%), and fats (21.3%), respectively [7]. A study by Fitzgerald et al. also reported that 79% of adults had ER [11]. It seems that ER is much lower in Iranian adults than in adults in other communities. The discrepancy might not only be attributable to differences in gender and sample size, but might also be related to the cultural and social factors affecting food-related beliefs and behaviors, ideal body appearance, and levels of food literacy in different communities. Although food quantity restrictions, especially on high-fat foods, may confer health benefits, self-imposed restrictions on food groups without medical supervision may accompany health risks [55].

It is noteworthy that, severe ER in food groups such as bread and cereals, seafood and dairy products, fruits, eggs, vegetables, and nuts may lead to health problems [7, 11]. Therefore, it is necessary to screen adults for the probability of ER in different food groups and its negative effects, such as impaired social functioning and weight loss [11] and to plan dietary recommendations and timely measures to prevent these problems. Our findings showed that the mean ER score was higher in men than in women. This finding contradicts the results of Rolls et al., who reported that women were less satisfied with their weight and body shape than men and therefore imposed more ER on themselves in many food groups [56].

The current study showed that almost half of the participants had FI, with the highest mean score for the item "I try to buy satisfactory food. "Mean scores for FI and its subscales were also higher in women than in men. Other studies have also shown that women are more likely than men to experience food-related involvement because they like fattening foods but think they should avoid them [19, 56, 57]. In an earlier study, the highest FI score was related to the item “satisfaction for preparing and cooking food for others” [57], which is not consistent with our results.

Most of our participants used less healthy food groups (pHDI-15) and low glycemic foods (lGIDI-4). In addition, lean, overweight, and obese subjects consumed more nHDI-16, hGIDI-7, hSDI-4, and hSFDI- 6 than those with a normal BMI. In the present study, among the FCC items, the item "How often do you consume whole cereals such as oats, barley, and wheat (whole or oatmeal), corn, popcorn, and brown rice?" received the lowest score, while the item "How often do you use white bread (lavash) or bakery products such as baguettes and toast?" received the highest score. Women also consumed more healthy food groups (pHDI-15 and lGIDI-4) than men. A previous study also reported that women scored higher on pHDI foods and lower on nHDI foods than men [58].

Although the current nutritional guidelines for the general population require more intake of food groups classified as healthy, such as pHDI-15 and lGIDI-4 [46], and less consumption of foods classified in unhealthy food groups (nHDI-16) and high GI (hGIDI-7) (such as white bread, white rice, plain pasta or cereal, fast foods, butter, pork, cheese, sausage, red meat, sweets, canned meat, carbonated and non-carbonated soft drinks, energetic drinks, and alcoholic beverages), but the consumption of unhealthy food groups such as nHDI-16, hSFDI-6, hSDI-4, and hGIDI-7 was common and at a moderate level among the participants in the present study, which is a warning for public health policymakers. This finding shows that adults in the northern regions of Iran are more likely to consume unhealthy food groups.

As suggested by Sobhani et al., it is necessary to change the diet of Iranian adults towards a healthy and sustainable DP by increasing the intake of dairy products, fruits, vegetables, grains, chicken, and legumes and reducing the consumption of bread, rice, pasta, and red meat, eggs, fats, sugar, and sweets [59]. This shift can be induced by implementing educational programs and changing advertising policies in the food industry. Our findings highlight the need to make DP healthy, especially in northern Iran. This is an urgent issue as the dietary culture is changing rapidly and extensively due to tourism in these regions and the influence of the dietary cultures of other societies. The touristic nature of these areas has made many restaurants that serve fast food accessible to local people. Meanwhile, most of the cities in northern Iran are located on the margin of the Caspian Sea, and the food culture in these regions was once rich in brown rice, fish, olive oil, olives, and vegetables. Therefore, it is necessary to explore what caused these people to switch from healthy to unhealthy food groups. It is possible to promote the adoption of healthy foods by offering organic foods at low prices in the local market. Community education through local and national media and the development of educational and counseling services for adults, especially males, through health service providers will help achieve this goal.

Nearly half of our participants received insufficient NK scores, and men had higher mean NK scores than women. Other studies have also reported moderate or weak NK levels in Iranian adults [60, 61]. However, our findings do not match those of Saeidlou et al., who reported that more than 50% of Iranian adults knew the food groups [62]. A lack of knowledge about the importance of a balanced diet may lead to inappropriate eating habits [63, 64]. Therefore, educational programs need to be developed to increase NK levels and promote healthy eating habits in adults, especially women. Such programs help people better understand dietary guidelines and motivate them to choose healthy food groups.

We found a negative association between ER and pHDI-15. Galinski et al. also found that ER, especially restricting undesirable foods, was associated with health-promoting eating behaviors in Polish girls [7]. Wadolowska et al. also reported that girls who did not limit their frequent consumption of fruits, vegetables, and beans, but restricted high-fat, sugar, and starch foods, had healthier eating behaviors [64]. Jezewska-Zychowicz et al. recommend further investigation into the behavioral causes and nutritional implications of ER [34]. On the other hand, ER can lead to unhealthy eating habits and eating disorders [65]. However, moderate restrictions on high-calorie and undesirable foods may be wise and beneficial for a healthy diet [15].

We also found an inverse correlation between ER and FI. Other studies have also shown that strong food involvement is directly related to the diversity and frequency of consumption among food groups. FI seems to have an indirect effect on the intake of various food groups by giving importance to health [66, 67].

In the present study, we found an inverse correlation between ER and NK. Yahia et al. also found an inverse association between NK and saturated fat and cholesterol intake. For example, people who received 300 mg of cholesterol per day had lower mean NK scores (8 and 7.9 points lower, respectively) than those with lower fat or cholesterol intake [63]. People’s NK seems to affect their eating habits and healthier food choices. The low NK scores of the majority of our participants signify the importance of nutrition education. Healthcare systems, virtual networks, and educational systems such as universities can play a prominent role in increasing NK levels and promoting healthy eating patterns among adults.

We also found a direct association between FI and pHDI-15. In this regard, Derinalp Çanakçı wrote: People with higher FI scores are more concerned with healthy eating and are more likely to seek out new healthy foods and change their diet [68]. Bell and Marshall also reported that people who were more involved in food were better able to distinguish between healthy and unhealthy foods [19]. A study by Alencar et al. also showed a direct relationship between the consumption of healthy foods such as fruits and vegetables and FI. The latter authors, citing Castro, also wrote: To achieve more FI, people should be motivated through strategies in five economic, biological, human rights, socio-cultural, and environmental factors [57]. However, Jezewska-Zychowicz showed that people with a higher FI had a healthier DP than those with a low FI [34].

In this study, we found a direct association between NK and FI. Medoro et al. also showed that people with a higher FI had more NK [17]. In the current study, pHDI-15 and LGIDI-4 indices were higher in individuals with normal BMI than in others. However, Kurnik-Łucka et al. found no significant relationship between BMI and intaking healthy food groups (pHDI) [58].

The present study showed that FI plays a mediating role in the relationship between ER and DP. This finding shows that the relationship between ER and DP can be explained to some extent by considering a third variable, such as FI. In other words, our results showed that people with low ER had more FI and a healthier diet than those with high ER. Our results highlight the need for programs that increase FI to increase the effectiveness of interventions to reduce ER in unhealthy food groups and interventions to promote healthy dietary patterns (pHDI-15). These results are consistent with the hypothesis of Jezewska-Zychowicz that more FI is associated with less ER and healthier DP. However, the results of the Jezewska-Zychowicz study show that people with a high FI have a higher ER and healthier DP across different food groups than those with a low FI [34]. Our results also showed that FI plays a mediating role in the relationship between NK and DP (pHDI_15 and nHDI_16). This finding indicates that people with more NK had more FI and a healthier DP compared to those with less NK. Therefore, actions to increase FI are recommended to increase FI, increase the effectiveness of educational interventions, increase NK, and help adults adopt a healthy DP.

## Conclusion

In this study, we discuss the relationship between ER and DP, and the role of FI as a mediating variable that can explain the relationship between NK and DP. The study identified seven main DPs among adult Iranians. The results showed that most participants ate small portions of the healthy food groups (pHDI-15) and low-glycemic foods (lGIDI-4). Therefore, there is an urgent need to change DP from unhealthy to healthy, especially in the northern regions of Iran. Adult men in Iran have less access to education and counseling at health centers than women, and policymakers need to take action to improve this situation. Our study showed that FI plays a mediating role in the relationship between NK and ER with DP. Therefore, it is recommended to pay attention to the role of FI as a mediating variable in interventions based on reducing ER, increasing NK, and transitioning from unhealthy to healthy DP.

### Strengths of the study

Our study is the first to investigate the mediating role of FI in the relationship between ER and DP in Iranian adults. In addition, for the first time, we examined the seven main DPs, including grain products, fruits/vegetables/legumes/potatoes, dairy products, meat/fish/eggs, fats, beverages, sweets and other products, among adults living in the northern regions of Iran. We also measured six food indices, including pro-Healthy-Diet-Index (pHDI-15, non-Healthy Diet-Index (nHDI-16), hGIDI-7, lGIDI-4, hSDI-4, and hSFDI-6. We compared the values of these indices in four BMI groups. We also calculated the DQS and measured the values of FI, ER, NK, and anthropometric indices. The use of culturally appropriate instruments, the large sample size, and the selection of adults with a variety of social and economic characteristics are among the other strengths of this study that increase the generalizability of the findings.

### Limitations

Although the study achieved its objectives, it may have some limitations. Self-reported assessments of DP may be subject to social desirability bias. The length of the tools may have exhausted the participants and prevented them from completing the questionnaires accurately. Conducting the study on healthy people and only in one of the northern provinces of Iran may limit the generalizability of the results.

## Supporting information

S1 Data All data files are available from the Zenodo database. (XLSX)
